# A recurrent truncating germline mutation in the *BRIP1*/*FANCJ* gene and susceptibility to prostate cancer

**DOI:** 10.1038/sj.bjc.6604847

**Published:** 2009-01-06

**Authors:** Z Kote-Jarai, S Jugurnauth, S Mulholland, D A Leongamornlert, M Guy, S Edwards, M Tymrakiewitcz, L O'Brien, A Hall, R Wilkinson, A A Al Olama, J Morrison, K Muir, D Neal, J Donovan, F Hamdy, D F Easton, The UKGPCS Collaborators, The British Association of Urological Surgeons' Section of Oncology, R Eeles

**Affiliations:** 1 Translational Cancer Genetics Team, The Institute of Cancer Research, 15 Cotswold Road, Sutton, Surrey SM2 5NG, UK; 2 The Royal Marsden NHS Foundation Trust, Downs Road, Sutton, Surrey SM2 5PT, and Fulham Road, London SW3 6JJ, UK; 3 CR-UK Genetic Epidemiology Unit, University of Cambridge, Strangeways Laboratory, Worts Causeway, Cambridge CB1 8RN, UK; 4 University of Nottingham Medical School, Queens Medical Centre, Nottingham NG7 2UH, UK; 5 Surgical Oncology (Uro-Oncology: S4), Departments of Oncology and Surgery, University of Cambridge, Box 279, Addenbrooke's Hospital, Hills Road, Cambridge CB2 2QQ, UK; 6 Cancer Research UK, Cambridge Research Institute, Li Ka Shing Centre, Robinson Way, Cambridge CB2 0RE, UK; 7 Department of Social Medicine, University of Bristol, Canynge Hall, Whiteladies Road, Bristol BS8 2PR, UK; 8 Academic Urology Unit, University of Sheffield, Sheffield S10 2JF, UK

**Keywords:** prostate cancer predisposition, *FANCJ/BRIP1*, deleterious mutation, SNPs

## Abstract

Although prostate cancer (PrCa) is one of the most common cancers in men in Western countries, little is known about the inherited factors that influence PrCa risk. On the basis of the fact that BRIP1/FANCJ interacts with BRCA1 and functions as a regulator of DNA double-strand break repair pathways, and that germline mutations within the *BRIP1/FANCJ* gene predispose to breast cancer, we chose this gene as a candidate for mutation screening in familial and young-onset PrCa cases. We identified a truncating mutation, R798X, in the *BRIP1/FANCJ* gene in 4 out of 2714 UK PrCa cases enriched for familial (2 out of 641; 0.3%) and young-onset cases (2 out of 2073; 0.1%). On screening 2045 controls from the UK population, we found one R798X sequence alteration (0.05%; odds ratio 2.4 (95% CI 0.25–23.4)). In addition, using our data from a genome-wide association study, we analysed 25 SNPs in the genomic region of the *BRIP1*/*FANCJ* gene. Two SNPs showed evidence of association with familial and young-onset PrCa (rs6504074; *P*_trend_=0.04 and rs8076727; *P*_trend_=0.01). These results suggest that truncating mutations in *BRIP1*/*FANCJ* might confer an increased risk of PrCa and common SNPs might also contribute to the alteration of risk, but larger case–control series will be required to confirm or refute this association.

Prostate cancer (PrCa) aggregates in families, in a consistent manner with an important inherited component (reviewed in Edwards and Eeles, 2004). The genetic components underlying this familial risk have, however, proved difficult to identify. Genetic studies using linkage analysis in high-risk families have identified several possible susceptibility loci (Tavtigian *et al*, 2001; Carpten *et al*, 2002), but none of them have been definitively established (Easton *et al*, 2003). It seems likely now that susceptibility to PrCa is mediated, at least partially, through a combination of multiple low-penetrance loci (Haiman *et al*, 2007; Eeles *et al*, 2008; Thomas *et al*, 2008).

Genome-wide association studies (GWAS) have identified common variants in several regions that are associated with PrCa risk (Eeles *et al*, 2008; Gudmundsson *et al*, 2008; Thomas *et al*, 2008). In addition, resequencing of candidate genes, notably those involved in DNA double-strand break repair, has identified rarer variants associated with a more substantial risk. The most important of these is *BRCA2* mutations, which confer a risk of approximately five-fold, but there is evidence that truncating mutations in *NBS1* and *CHEK2* also confer susceptibility to PrCa (Dong *et al*, 2003; Edwards *et al*, 2003; Cybulski *et al*, 2004; Agalliu *et al*, 2007).

The *BRIP1*/*FANCJ* gene encodes a helicase in which the C-terminal domain is reported to interact with BRCA1 (Cantor *et al*, 2001). The *BRIP1* gene spans 180 kb, comprises 20 exons and encodes a protein of 1249 amino acids. It is located on chromosome 17q22, distal to *BRCA1*, which is at 17q21. BRIP1/FANCJ has an important role in BRCA-associated DNA damage repair functions, works as a DNA-dependent ATPase and a DNA helicase, and is essential for DNA repair and genomic stability. It forms a complex with the BRCT domain of BRCA1, and this is important for the role of BRCA1 in its DNA double-strand break repair function (Levitus *et al*, 2006).

Germline mutations in *BRIP1/FANCJ* are associated with Fanconi anaemia, a chromosomal instability syndrome characterised by developmental abnormalities and predisposition to cancer (Levitus *et al*, 2005). Truncating mutations in the *BRIP1/FANCJ* gene have recently been shown to be associated with a moderate risk of breast cancer (Seal *et al*, 2006). Given the observed associations of PrCa with other DNA repair genes, we considered *BRIP1/FANCJ* as an attractive candidate for PrCa-predisposition gene.

## Materials and methods

Whole blood samples from PrCa cases were collected as part of the UK Genetic Prostate Cancer Study (UKGPCS), a national study of familial and young cases of PrCa. Familial samples were from 192 cases with two affected relatives (‘strong family history’) and 449 other samples with one affected relative (‘some family history’). One person per family was used for sequence analysis. Wherever possible, the youngest affected family member was considered. Young-onset cases comprised 2073 men with a diagnosis of PrCa at age ⩽60 years. All self-reported non-Caucasians were excluded. The study was approved by the multiregional ethics committee and all patients gave informed consent. All PrCa diagnosis was based on the verification of the histological report or death certificate.

Caucasian controls comprised 2045 blood DNA samples, which were selected through the ProtecT study. ProtecT is a national study of community-based PSA testing and a randomised trial of subsequent PrCa treatment. Men between the ages of 50 and 69 years are recruited through general practices in nine centres in the UK. Ethnically matched controls were sampled from these centres to match approximately the geographical distribution of the cases. DNA was extracted using standard methods as described in Eeles *et al* (2008).

The full coding sequence and exon–intron boundaries of the 192 familial samples were analysed by sequencing. The sequencing was carried out using the BigDyeTerminator v3.2 Cycle Sequencing kit and the 3700 automated sequencer (Applied Biosystems, Foster City, CA, USA).

All the additional samples, 2073 young-onset cases and 2045 UK population controls were tested for the R798X sequence variant by the 5′nuclease assay (Taqman™) using the ABIPrism 7900HT sequence detection system according to the manufacturer's instructions. Primers and probes were supplied directly by Applied Biosystems (http://www.appliedbiosystems.com/) as Assays-By-Design™.

We had earlier carried out a GWAS, involving 1854 of the PrCa cases and 1894 controls using the Illumina Infinium HumanHap550 array (Eeles *et al*, 2008). From these data, we identified 25 SNPs from the 270-kb region covering the genomic sequences of *BRIP1/FANCJ,* which were used to obtain evidence for associations between common variants and PrCa risk.

Significance tests, odds ratios (ORs) and confidence limits were computed using standard methods. For the SNPs, both Cochran–Armitage trend tests and 2-degrees-of-freedom tests were calculated. For Hardy–Weinberg equilibrium and Armitage trend testing, we used the public software developed by Tim M Strom and Thomas F Wienker (http://ihg.gsf.de/cgi-bin/hw/hwa1.pl).

## Results

**Figure 1:**
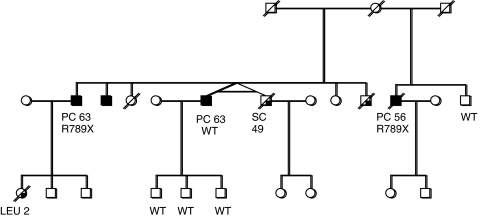
Prostate cancer (PC) family with the R798X nonsense mutation in *BRIP1/FANCJ.* Wild-type (WT) labels on individuals tested but non-carriers of R798X, Leu=leukaemia, SC=stomach cancer. (The pedigree has been modified to maintain confidentiality.)

Initially, a set of genomic DNA samples representing the youngest affected individual from 192 multiple case PrCa families was screened. The full coding sequence and exon–intron boundaries of the *BRIP1*/*FANCJ* were analysed by sequencing. A truncating mutation, R798X (1 out of 192, 0.52%) was identified in the proband from a family with three affected brothers (Table 1). The pedigree and the result of the segregation analysis are shown in Figure 1. The available blood samples were tested for this germline mutation and one affected brother carried the mutation, whereas the unaffected brother did not. Only a paraffin-embedded tissue sample (biopsy) was available from the third affected brother, and sequencing the DNA sample from this did not show the mutation. Blood samples from his three sons were also tested and were all found to be non-carriers for the R798X mutation. In addition to this deleterious mutation, several missense mutations were identified in the 192 familial samples, as presented in Table 2. No other mutations that were predicted to truncate the protein were identified.

To establish the contribution of the *BRIP1*/*FANCJ* R798X mutation to PrCa predisposition, we screened a further 449 samples with a single affected relative and 2073 young-onset PrCa cases, (individuals diagnosed with PrCa before 60 years of age with no family history) and 2045 controls. The R798X mutation was present in two of the young-onset cases (0.10%), in one of the familial cases (0.22%), and in one of the controls (0.05%) (Table 1). The ages of onset of the mutation carriers were 49 and 60 years. Neither case had a family history of PrCa. However, one of these mutation carriers had a family history of other cancers and malformations, which appear characteristic of Gorlin syndrome. The familial case had one affected second-degree relative with an unknown age of onset, and also had relatives affected with other cancers (colon and stomach cancer).

On the basis of our data, the estimated OR for PrCa was 2.4 (95% CI 0.25–23.4). If the control data of Seal *et al* (2006) are also considered in our analysis, the estimate is somewhat more precise (OR 2.5, 95% CI 0.41–14.7) but still not significantly different from 1.

We found five other amino-acid substitutions in the 192 familial samples sequenced (Table 2). All have been reported earlier (Seal *et al*, 2006; Guénard *et al*, 2008). All of these sequence variants were also found in the control series. The common variant p.Pro919Ser showed some evidence of an association with risk (*P*_trend_=0.034) in this set of cases with strong family history. However, this SNP (rs4986764) was also included on the Illumina 550k array used in our GWAS (Eeles *et al*, 2008). In this larger study of familial and young-onset cases there was no evidence of association with PrCa (*P*_trend_=0.48). None of the four rare variants showed a difference in frequency between cases and controls.

We also analysed the genotype frequencies of 25 SNPs from the Illumina 550k array in the genomic region of *FANCJ/BRIP1* for 1853 PrCa cases and 1880 control individuals (Eeles *et al*, 2008). We found significant differences in genotype frequencies between cases and controls for two SNPs: rs6504074 and rs8076727 (Table 3). The estimated per allele OR for rs6504074 was 1.10 (*P*_trend_=0.029); this rose to 1.20 (*P*_trend_=0.01) when the analysis was restricted to familial cases. The estimated OR for rs8076727 was 1.29 (*P*=0.01); this rose to 1.39 (*P*=0.01) for familial cases.

## Discussion

We have identified a recurrent deleterious mutation in the *BRIP1/FANCJ* gene in a set of familial and young-onset PrCa cases.

This truncating mutation has been reported earlier in four Fanconi anaemia subtype J families of diverse geographic origin (Levitus *et al*, 2005) and in five breast cancer families (Seal *et al*, 2006), suggesting that it is a relatively ancient founder mutation. The frequency of this mutation in our control population is almost identical to that found in the study by Seal *et al* (2006) (1 in 2081).

This recurrent R798X mutation in exon 17 predicts a truncated protein in which the helicase motif VI and the BRCA1 interacting regions are deleted. BRIP1/FANCJ binds directly to the C-terminal BRCA repeats of BRCA1 and this interaction is regulated in a cell-cycle-dependent manner. However, in the Fanconi anaemia pathway for DNA crosslink repair, BRIP1/FANCJ functions independently of this interaction (Bridge *et al*, 2005).

*BRIP1*/*FANCJ* encodes a DEAH-box helicase and can directly interact with DNA (Bridge *et al*, 2005). It can unwind DNA structures such as Holliday junctions formed during homologous recombination, suggesting an association with some DNA repair processes (Levitus *et al*, 2006). It is interesting that most of the Fanconi anaemia genes encode proteins that form a nuclear core complex with a function of monoubiquitination of FANCD2, but FANCD1 (BRCA2) and BRIP1/FANCJ function downstream of this and they both have a more defined role in DNA damage repair.

We have reported earlier that 2% of men diagnosed with PrCa at <55 years had a germline mutation in the *BRCA2* gene (Edwards *et al*, 2003)*. BRCA2* is a high-risk breast cancer predisposition gene and is also known as *FANCD1*, responsible for causing FA-D1. It is therefore plausible that truncating mutations in *BRID1/FANCJ* also predispose to PrCa. Our results for the R798X mutation are consistent with a moderate risk of PrCa, comparable to that for breast cancer, but larger numbers will be required to confirm this.

We found five other amino-acid substitutions in the 192 familial samples sequenced (Table 2), all of which have been reported earlier (Seal *et al*, 2006; Guénard *et al*, 2008). Three of these variants are situated within the Rad3-related DNA helicase domain (res.1–888) and one, the common p.Pro919Ser variant, is within the BRCA1-binding domain (Bridge *et al*, 2005). The p.Arg173 is a strongly conserved residue in mammals, whereas the other variants are poorly conserved in other species (Guénard *et al*, 2008). We found no evidence for significant association between these sequence variants and PrCa risk.

We have also evaluated the association between 25 common SNPs and the risk of PrCa in a case–control design and found two SNPs significantly associated with PrCa risk. Both of the associated SNPs are in intron 6. These associations, although nominally significant, do not reach the stringent levels of significance appropriate for candidate gene or genome-wide scans, and could have occurred by chance. However, if verified in larger series, they could be markers for common variants in *BRID1/FANCJ* influencing the risk.

In conclusion, we report that deleterious mutations in *BRIP1/FANCJ* occur in a modest number of familial and young-onset PrCa cases. In addition, we found evidence of association with two common SNPs. These observations warrant further evaluation in independent case–control studies.

## Figures and Tables

**Table 1:** Recurring mutation in *BRIP1*/*FANCJ* in prostate cancer

**Mutation**	**Study group**	**No. of samples screened**	**No. of mutations**	**Frequency**	***P*-value**
p.Arg798Stop R798X	Cases with strong family history (proband from families of three or more PrCa)	192	1	0.52 %	0.16
	Cases with some family history (proband from families of two PrCa)	449	1	0.22%	0.33
	Young-onset cases (diagnosed at ⩽60 years of age)	2073	2	0.10%	0.51
	All young-onset or familial PrCa tested	2714	4	0.14%	0.29
	UK population controls	2045	1	0.05%	

PrCa=prostate cancer.

**Table 2:** Non-synonymous sequence variants found in *BRIP1*/*FANCJ* in familial PrCa

					**Cases, *n* (%)**	**Controls^a^, *n* (%)**
**Exon**	**Nucleotide change**	**dbSNP ID**	**Protein change**		***n*=192**	***n*=2081**
6	c.517C>T	rs4988345	p.Arg173Cys		1 (0.52%)	24 (1.10%)
	c.577G>A	rs4988346	p.Val193Ile		1 (0.52%)	14 (0.67%)
	c.584T>C		p.Leu195Pro		2 (1.04%)	6 (0.28%)
7	c.890A>G		p.Lys297Arg		2 (1.04%)	6 (0.28%)
19	c.2755C>T	rs4986764	p.Pro919Ser	Pro/Ser	83 (43.00%)	970 (46.0%)
				Ser/Ser	44 (22.9%)	328 (15.7%)

PrCa=prostate cancer.

a Data from Seal *et al* (2006).

**Table 3:** Prostate cancer genotype specific risk for two SNPs in *BRIP1*/*FANCJ* genomic region

	**GG**	**TG**	**TT**	**Per allele OR (95% CI)**	**OR Hom (95% CI)**	**OR Het (95% CI)**	***P*_Het_**	***P*_trend_**
*rs6504074*								
Familial	345	274	64	1.20 (1.04–1.37)	1.57 (1.13–2.17)	1.13 (0.94–1.35)	0.21	0.01
Young	618	453	87	1.07 (0.94–1.20)	1.19 (0.89–1.59)	1.04 (0.89–1.21)	0.62	0.28
All	963	727	151	1.11 (1.00–1.23)	1.33 (1.03–1.71)	1.07 (0.94–1.23)	0.32	0.04
Control	1031	727	122					
								
*rs8076727*								
Familial	373	261	46	1.20 (1.04–1.38)	1.39 (0.96–2.00)	1.21 (1.01–1.46)	0.04	0.01
Young	658	432	72	1.12 (1.00–1.27)	1.23 (0.90–1.69)	1.14 (0.98–1.33)	0.10	0.06
All	1031	693	118	1.13 (1.03–1.28)	1.29 (0.97–1.70)	1.17 (1.017–1.336)	0.03	0.01
Control	1124	648	100					

OR=odds ratio; SNP=single nucleotide polymorphism.
